# A COVID-19 Vaccine: Big Strides Come with Big Challenges

**DOI:** 10.3390/vaccines9010039

**Published:** 2021-01-11

**Authors:** Juanita Mellet, Michael S. Pepper

**Affiliations:** SAMRC Extramural Unit for Stem Cell Research and Therapy, Department of Immunology, Faculty of Health Sciences, Institute for Cellular and Molecular Medicine, University of Pretoria, Pretoria 0001, South Africa; juanitamellet@yahoo.co.uk

**Keywords:** COVID-19, SARS-CoV-2, vaccine, vaccine development, vaccine platforms, immune response

## Abstract

As of 8 January 2021, there were 86,749,940 confirmed coronavirus disease 2019 (COVID-19) cases and 1,890,342 COVID-19-related deaths worldwide, as reported by the World Health Organization (WHO). In order to address the COVID-19 pandemic by limiting transmission, an intense global effort is underway to develop a vaccine against SARS-CoV-2. The development of a safe and effective vaccine usually requires several years of pre-clinical and clinical stages of evaluation and requires strict regulatory approvals before it can be manufactured in bulk and distributed. Since the global impact of COVID-19 is unprecedented in the modern era, the development and testing of a new vaccine are being expedited. Given the high-level of attrition during vaccine development, simultaneous testing of multiple candidates increases the probability of finding one that is effective. Over 200 vaccines are currently in development, with over 60 candidate vaccines being tested in clinical trials. These make use of various platforms and are at different stages of development. This review discusses the different phases of vaccine development and the various platforms in use for candidate COVID-19 vaccines, including their progress to date. The potential challenges once a vaccine becomes available are also addressed.

## 1. Introduction

Coronavirus disease 2019 (COVID-19) caused by severe acute respiratory syndrome-coronavirus-2 (SARS-CoV-2) was responsible for 86,749,940 reported infections and 1,890,342 reported deaths as of 8 January 2021, as indicated by the World Health Organization (WHO) [1]. An effective vaccine is urgently needed to control the pandemic and to prevent future outbreaks. Due to the novelty of the virus, there are currently few approved treatments for COVID-19 and a limited number of vaccines have only recently been approved for SARS-CoV-2. Vaccines have been effective in protecting against several deadly diseases and at present prevent roughly 2.5 million deaths per year [2,3]. Although their mechanism of action is complex and not completely understood, this involves both innate and adaptive immunity as they prime the immune system to respond to invading pathogens [4]. Following initial exposure to an antigen, the immune system is activated in a primary immune response, where pathogens are recognized by pattern recognition receptors (PRRs) directed against evolutionarily conserved pathogen-associated products that are not contained in self-antigens [5]. This leads to secretion of various signaling molecules including interferon gamma (IFN-γ) to initiate and direct innate and adaptive antiviral immune responses. Specific cell types such as natural killer (NK) and dendritic cells (DCs) are recruited to sites of inflammation. When DCs encounter pathogens they undergo rapid maturation, modulate specific cell surface receptors, and secrete additional cytokines and chemokines. IFN receptor signaling is essential for DC maturation and migration to secondary lymph nodes, where they provide co-stimulatory signals to initiate antiviral B- and T-cell responses [5]. B- and T-cells bind to viral proteins through antigen receptors leading to activation, expansion, differentiation and secretion of effector molecules to assist in controlling the infection. Once the infection clears, approximately 90% of the virus-specific cells die, while 10% persist as long-lived memory cells. These memory cells can produce a continuous supply of effector molecules in response to reinfection [6].

The purpose of vaccines is to initiate a primary immune response by introducing altered or weakened antigens (or parts thereof) that usually cause disease, in order to develop immune memory without the host becoming infected naturally [4]. Vaccines should ideally trigger B- and T-cell responses. Vaccine efficacy is primarily conferred by inducing antigen-specific antibodies. The quality of the antibodies (affinity, specificity, and/or neutralizing capacity) is the determining factor in their efficacy. Persistence of vaccine antibodies above protective thresholds and/or the maintenance of immune memory cells capable of rapid and effective reactivation following subsequent exposure, are important for long-term protection [4]. Recent evidence suggests that vaccines induce not only disease-specific effects, but also favorable non-specific effects against unrelated pathogens [7].

## 2. Vaccine Platforms

**Figure 1:**
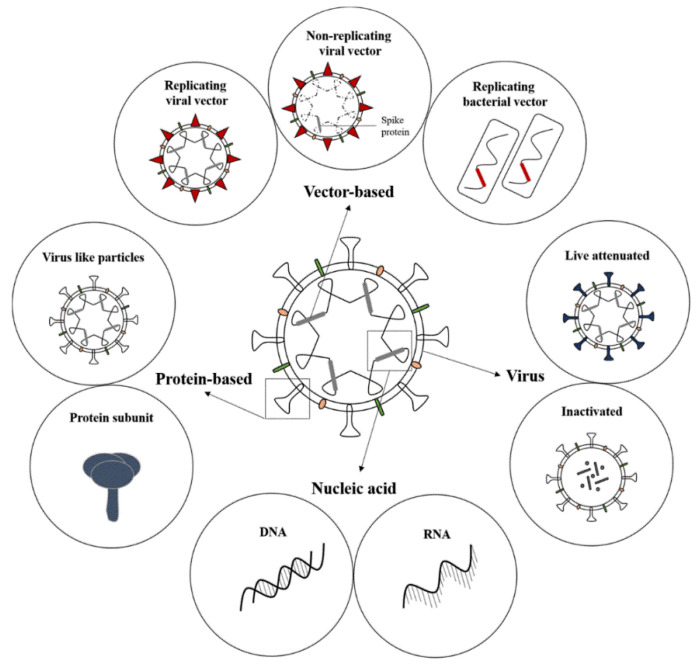
SARS-CoV-2 vaccine types. SARS-CoV-2 vaccines currently in pre-clinical and clinical evaluation make use of various platforms (image created by Juanita Mellet).

**Figure 2:**
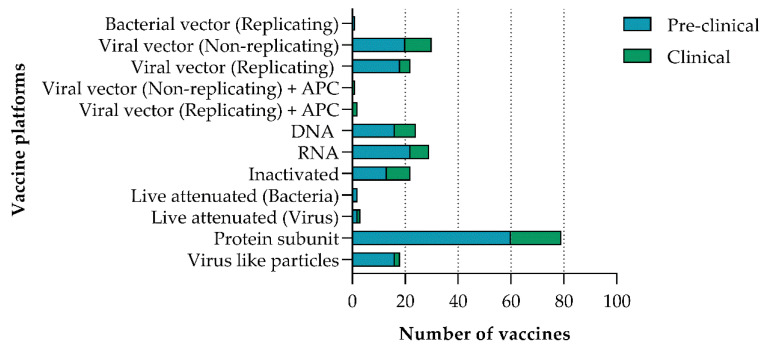
Platforms in use for COVID-19 candidate vaccine development in pre-clinical (blue) and clinical (green) trials [9]. APC: antigen presenting cell; DNA: deoxyribonucleic acid; RNA: ribonucleic acid. (Data figure created by Juanita Mellet.)

The initial assessment of vaccines relied on observing the response of the recipient, but this has been replaced by advanced technologies that have allowed vaccines to be more specific and safer [8]. Vaccines against SARS-CoV-2 are currently being evaluated at pre-clinical and clinical levels that make use of 12 different platforms. Multiple vaccine types exist such as nucleic acid-, viral vector-, virus-, and protein subunits, to name a few (Figure 1). Over 70 vaccines are being evaluated that use the protein subunit platform, 30 use non-replicating viral vector platforms and 29 use RNA platforms (Figure 2). In total, 10 different vaccine types are being evaluated in clinical trials, with the most frequent being protein subunits, non-replicating viral vectors, inactivated viruses, and viral DNA (Figure 2) [9].

Purified inactivated (or killed) viruses have been used traditionally for vaccine development and consist of chemically inactivated versions that are incapable of causing disease [10]. Inactivated vaccines have been found to be safe and effective for the prevention of diseases caused by viruses such as influenza [10]. Protein subunit vaccines use specific pieces of SARS-CoV-2 to initiate an immune response that is targeted to key parts of the virus [10]. A major benefit of this type of vaccine is that it can be used on everyone, including people with weakened immune systems and long-term health problems. These vaccines are popular due to their wide application and proven safety, which is most likely why there are over 70 candidate vaccines in pre-clinical and clinical testing, making use of this platform (Figure 2). A limitation of this type of vaccine is that individuals might require booster shots for continuous protection against a particular pathogen [10]. Protein subunit vaccines are used to protect against hepatitis B, human papilloma virus, and meningococcal disease, to name a few [10]. To date, there are no approved RNA vaccines in humans [11,12]; however, seven candidate COVID-19 RNA vaccines are being evaluated in clinical trials (Figure 2), with one having recently been approved in several countries and another approved for emergency use in the United States (US) [10]. RNA vaccines are especially attractive since they are easier and faster to manufacture in large quantities [11]. Replicating/non-replicating viral vector vaccines make use of non-SARS-CoV-2 viruses (dead or weakened) and include genetic material from SARS-CoV-2 to which the immune system will respond. Several vaccines make use of non-replicating adenoviruses (Ad). Ad-based vaccines are safe and have previously been used in immunocompromised individuals [13] who are considered to be at higher risk for developing severe COVID-19 [14]. Another advantage of Ad-based vaccines is that they need to be kept cold rather than frozen, which makes distribution easier. Several Ad-based vaccines are being evaluated in clinical trials. Two make use of adenovirus type 5 (Ad5) vectors that are based on a common cold virus. In total, 40% of people in the US and 90% of people in sub-Saharan Africa have pre-existing antibodies from natural exposure to Ad5 [15], which could limit the effectiveness of such a vaccine [16]. Studies on pre-existing immunity have revealed that neutralizing antibodies, Ad-specific T-cells and adenovirus induced inflammatory responses contribute to reduced vector efficacy [17,18]. Studies that have assessed both neutralizing antibodies and T-cell responses to Ad5 have shown that a higher proportion of individuals possesses T-cell immunity compared to neutralizing antibodies [19,20]. Ad26, a less common adenovirus, is used in vaccine candidate Ad26.COV2.S and a combination of Ad26 and Ad5 is used in Sputnik V [21,22]. Ad26 neutralizing antibodies are common in adults in sub-Saharan Africa and southeast Asia, but lower titers are observed when compared to Ad5, and Ad26 neutralizing antibodies did not suppress the immunogenicity of an rAd26-based vaccine in rhesus monkeys [23]. A chimpanzee adenovirus is used in the ChAdOx1/AZD1222/Covishield vaccine being assessed in clinical trials. Antibodies to chimpanzee adenoviruses are uncommon in the US, but more prevalent in people in sub-Saharan Africa [15].

## 3. Vaccine Development

**Figure 3:**
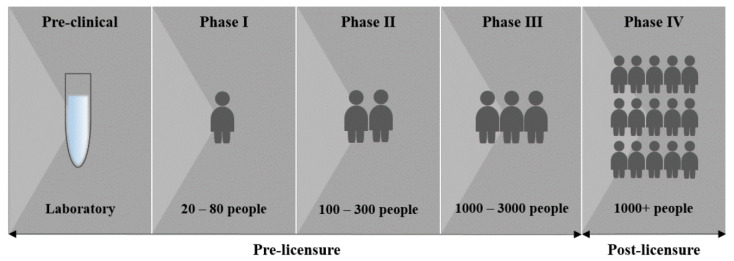
Phases of vaccine development (image created by Juanita Mellet).

The development of a safe and effective vaccine usually takes several years as it needs to proceed through clinical trials and requires strict regulatory approvals before it can be manufactured and distributed [24]. Since the COVID-19 pandemic is having a major impact globally, the processes of developing and testing a new vaccine are being expedited. Vaccines are usually first evaluated in a pre-clinical phase using either human/mammalian cell cultures (in vitro) or appropriate animal models (in vivo). Due to the novelty of the virus, the best suited animal model has yet to be determined. However, rhesus macaques, which are a non-human primate species that presents with a COVID-19-like disease upon infection with SARS-CoV-2, have been used as an animal model in some pre-clinical studies [13,25,26]. Pre-clinical evaluation of candidate vaccines is a prerequisite to initiating clinical trials. In the case of positive results in the pre-clinical phase, vaccine candidates proceed through testing over three clinical trial phases to determine whether they are safe and effective in humans (Figure 3). On average, the probability of a vaccine entering the market, taken from the pre-clinical phase through clinical testing and licensure, is below 10% [24].

The first phase involves testing candidate vaccines on a small number (20–80) of healthy individuals to ensure that the vaccine is not harmful and to determine efficacy. Tolerance and expected adverse effects due to the vaccine or vaccination process are evaluated in phase I trials [27]. To ensure that safety data from different sites is comparable, a standardized approach of data collection, analysis, and reporting is required [27]. Phase I also determines the optimal dosage through a dose escalation study, where groups of participants receive different escalating doses of the vaccine. This aids in determining the optimal dose for follow-up testing.

If favorable results are achieved in phase I, candidate vaccines proceed to phase II. The second phase involves testing the vaccine on a larger sample size (100–300) and is expected to provide clinically meaningful information on safety, immunogenicity and efficacy of the candidate vaccine [27]. This phase involves testing the vaccine on hundreds of individuals in the target population at multiple sites, allowing researchers to make conclusions on whether the vaccine is safe, results in the desired immune response, and confers protection. In some cases it might be necessary to conduct multiple phase II studies to address schedules, age group variations, and duration of follow-up, before proceeding to phase III [27].

Phase III is the most pivotal part of a study on which licensing is based. In this phase the vaccine is given to many people (1000–3000) in the target population and is tested for efficacy and safety. A single study may not be sufficient to address all questions and it may therefore be necessary to test the vaccine under different conditions including disease patterns and populations [27]. If the phase III results demonstrate efficacy and safety, the manufacturer can submit an application to license and market the vaccine to a national regulatory authority [27].

**Figure 4:**
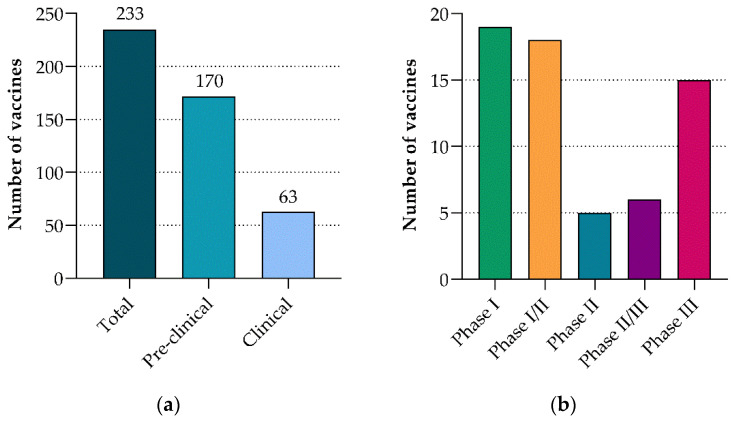
COVID-19 candidate vaccines. (**a**) The number of candidate COVID-19 vaccines in pre-clinical and clinical evaluation [9]. (**b**) The number of candidate COVID-19 vaccines in different phases of clinical evaluation [9]. (Data figures created by Juanita Mellet.)

As of 8 January 2021, there were 233 candidate vaccines in development for COVID-19, with 170 being evaluated in the pre-clinical and 63 in the clinical phases of testing (Figure 4a) [9]. The majority were in early phase clinical trials (phase I or I/II), while 15 vaccine candidates were in phase III (Figure 4b). The next section will focus on vaccine candidates with published data in advanced stages of development (phase III) (Table 1).

COVID-19 vaccines in phase III were initially evaluated in young healthy volunteers. Older participants and individuals with comorbidities at higher risk of developing severe disease were included once the vaccines were proven to be safe in healthy volunteers [29]. Some candidate vaccines in the later stages of development included different population groups across the globe, since dosage and effectiveness could vary depending on the population. In the US, COVID-19-related morbidity and mortality rates are higher among certain ethnic/racial groups [30,31,32], and it is important to include participants across a broad spectrum to determine whether candidate vaccines are safe and effective in these groups. According to the US Food and Drug Administration (FDA), vaccine participants have not necessarily been representative of all racial/ethnic groups [33].

### 3.1. Nucleic Acid Vaccines

The first vaccine entered human trials on 16 March 2020 and was an RNA-based vaccine, mRNA-1273, developed by Moderna and the National Institute of Allergy and Infectious Diseases (NIAID). Phase I results indicated that protective antibodies were produced in a group of 45 healthy volunteers. The levels of protective antibodies in participants were similar to those detected in recovered COVID-19 patients. Effectiveness and optimal dose were also determined during phase I [34]. A separate phase I study performed a dose-escalation in older adults (age 65 and older), the findings of which support the current dose being tested in phase III trials [28]. The next phase of testing involved 30,000 participants and included individuals aged between ages 18 and 55. Vaccine efficacy was 94.1% and showed protection for up to three months [35,36]. On 17 December 2020, this vaccine received approval from the FDA for emergency use in the US [37]. One healthcare worker experienced a serious allergic reaction after being vaccinated [38]. Moderna recently registered a trial to test the vaccine in adolescents, aged 12–17 [39].

BioNTech and Pfizer developed an RNA vaccine, BNT162b2. Initial phase I/II results involving 60 healthy volunteers aged between 18 and 55 years were encouraging. Broadly neutralizing antibodies were detected and CD4+ and CD8+ T-cell responses against the SARS-CoV-2 receptor binding domain (RBD) were observed at low doses. Phase III testing involved 30,000 healthy participants and showed 95% efficacy [40]. The vaccine caused frequent short-lived fatigue, fever and muscle aches, and four serious adverse events were reported [40]. Six vaccine participants died, although none of the deaths were ascribed to the vaccine. Efficacy was 52% after the initial dose which increased to 91% after the second dose [40]. The same level of protection was observed in individuals of different ages, different ethnic/racial groups and people with diabetes and/or obesity. These positive phase III results led to emergency approval in the US, United Kingdom (UK), Bahrain, Mexico, Singapore, and Canada [41]. Vaccine administration started on 8 December 2020 in the UK and millions of vaccines have been shipped to several countries worldwide [42]. Healthcare workers are among the first to have been vaccinated and eight experienced severe allergic reactions minutes after receiving the vaccine [43,44,45].

Both mRNA vaccines use a lipid-based nanoparticle (LNP) carrier system, which also acts as an adjuvant. The LNPs are stabilized with polyethylene glycol (PEG), prolonging their lifespan. Scientists speculate that these allergic reactions might be related to either the lipid or the PEG component of these vaccines [46].

### 3.2. Inactivated Vaccines

PicoVacc/CoronaVac, an inactivated COVID-19 vaccine developed by Sinovac, received approval on 3 July 2020 to proceed to phase III after promising pre-clinical results and the absence of serious adverse events in 743 volunteers in phase I/II testing [25].

Bharat Biotech in collaboration with the Indian Council of Medical Research (ICMR) recently registered a final phase testing protocol of candidate vaccine BBV152, which will take place at 25 centers in India. The plan is to launch this vaccine in the second quarter of 2021 once regulatory approval has been obtained.

### 3.3. Non-Replicating Viral Vector Vaccines

Four non-replicating viral vector vaccines have entered phase III [9]. Phase I/II results on Ad5-nCoV, developed by CanSino Biological Inc. and the Beijing Institute of Biotechnology, have been released [47,48]. A dose-escalating phase I trial tested three different doses (low, medium, and high). Low and medium were better tolerated, while higher doses resulted in a higher reactive profile that presented as severe fever, fatigue, and muscle and joint pain [48]. Low and medium doses were further evaluated in a larger phase II study. The low dose (5 × 10^10^ viral particles) vaccine showed better safety and immunogenicity [47]. It was noted that more than half of the participants had pre-existing immunity to Ad5 [47].

A non-replicating viral vector vaccine developed by the Gamaleya Research Institute of Epidemiology and Microbiology in Moscow was approved in Russia. At the time of registration, the vaccine had not yet entered the final testing phase in humans, and no results had been published on earlier clinical phase testing [49]. Scientists responded globally by saying that the registration was premature and inappropriate, as the vaccine could only be administered once efficacy had been proven in a larger cohort [50]. Initial results have since been published: the vaccine appears to be effective and no serious side effects have been reported [51]. Testing on a larger cohort has commenced and involves over 40,000 people across a number of countries [22]. Results from the double-blind, randomized, placebo-controlled phase III trial indicate >90% effectiveness in protecting against COVID-19 with no serious side effects [22]. The Gamaleya Research Institute recently partnered with AstraZeneca, which makes the adenovirus vaccine ChAdOx1/AZD1222/Covishield. The objective is to combine the vaccines to determine whether stronger protection can be achieved when the two are used together [52].

Janssen Pharmaceutica is using Ad26 to develop a COVID-19 vaccine. This single dose vaccine provided protection in monkeys and showed a promising safety profile and immunogenicity in phase I human trials [21]. A phase III trial was launched in September 2020, which was paused in October following an adverse reaction in a volunteer [53]. A second phase III trial was recently launched to assess the effect of two doses [54].

The COVID-19 Oxford vaccine (AZD1222) is a collaborative effort between the University of Oxford, the Oxford Jenner Institute and AstraZeneca. This vaccine makes use of a non-replicating chimpanzee adenovirus (common cold virus, ChAdOx1) that contains the genetic sequence of the SARS-CoV-2 surface spike (S) protein [13]. More than 1000 immunizations were performed in a phase I trial that involved various institutions across the UK [13]. No serious adverse events were recorded. The vaccine resulted in the production of neutralizing antibodies and also elicited a T-cell response [13]. More than 10,000 adults and children were enrolled in a phase II study to assess the immune response in people of different ages. It proved to be effective in people of all ages including young and older individuals (above age 70), suggesting that it will protect the most vulnerable [55]. The final stage tested the vaccine in 30,000 individuals; this was conducted in the UK, South Africa, Brazil, and the US [56,57]. Trials were halted in the US in September 2020 after a serious adverse reaction (transverse myelitis) occurred in one participant. In this individual, not previously diagnosed with multiple sclerosis, the transverse myelitis was found to be unrelated to the vaccine [58]. Trials have since resumed and recently published phase III results indicate up to 90% efficacy. Volunteers all received two doses, with some receiving a half-strength first dose. The half-strength dose led to 90% efficacy while the two standard doses led to 62% efficacy [59]. It later transpired that the lower initial dose, which resulted in a stronger immune response, had not originally been planned, but was the result of a mistake related to how the doses had been measured out. Lower doses were only assessed in volunteers younger than 55 [60].

### 3.4. Protein-Based Vaccines

The first protein subunit vaccine is being developed by Novavax and has entered phase III testing. Phase I results, which included 131 healthy adults, were recently published. This vaccine includes an adjuvant (Matrix-M1) in some groups that resulted in an enhanced immune response with acceptable safety. A second vaccination in the same individuals resulted in increased neutralizing antibody and T-helper-cell responses when the Matrix-M1 adjuvant was included. The levels observed in participants were equal to those seen in hospitalized patients with COVID-19 [61].

The first protein-based vaccine from China [62] recently commenced phase III trials and involves testing on 29,000 volunteers aged 18 and above. This vaccine is being developed by Anhui Zhifei Longcom Biopharmaceutical and the Institute of Microbiology, Chinese Academy of Sciences.

## 4. COVID-19 Vaccine Challenges

Despite efforts to develop a vaccine against SARS-CoV-2, experts warn that a vaccine might not completely eradicate this disease as levels of efficacy may vary or be incomplete, and not everyone will have access or wish to be vaccinated. As discussed below, there are several challenges that need to be addressed for a vaccine to achieve maximum effect.

### 4.1. Long-Term Immunity

Historical studies on the coronavirus family including severe acute respiratory syndrome (SARS) and Middle East respiratory syndrome (MERS) as well as an early study on SARS-CoV-2 suggested that infection does not provide long-term immunity [63,64]. However, a recent report found antibodies and memory B- and T-cells for up to three months in recovered COVID-19 patients [6]. Viral vaccines primarily use antibody-mediated effector mechanisms to prevent infection by binding directly to the virus before infection occurs [65]. However, cell-mediated responses are equally important and limit disease severity by destroying infected cells [65] and by producing long lasting memory T- and B-cells that will be essential for sustained antibody production. Longitudinal assessment of vaccine participants is therefore critical and provides information on whether long-lasting immunity can be achieved through vaccination, or whether immune booster therapy would be required every few months [34]. The use of adjuvants has previously been shown to improve long-term memory [66] and several candidate vaccines already in clinical trials make use of adjuvants [9]. If long-lasting immunity is not achieved, a vaccine could still protect vulnerable individuals and healthcare workers by reducing the amount of virus that a vaccinated person generates and emits [67].

### 4.2. Antibody-Dependent Enhancement (ADE) of Disease

Antibody-mediated protection is well described, and antibody detection is used to determine effectiveness of many human vaccines. However, past experience with other viruses suggests that antibodies might enhance inflammatory responses. This is termed antibody-dependent enhancement (ADE) of the disease, and is due to the presence of poorly neutralizing cross-reactive antibodies that bind to the virus and enhance viral entry into cells [68]. This is a concern for SARS-CoV-2 vaccine development since host antiviral responses could hypothetically become harmful post-vaccination [69]. A meta-analysis of COVID-19 patients treated with convalescent plasma from recovered COVID-19 patients that harbors circulating antibodies against SARS-CoV-2, resulted in minimal adverse events [70,71]. Low immunoglobulin G (IgG) antibody levels in transfused plasma were associated with higher mortality, and mortality decreased as plasma IgG levels increased [72]. This suggests that COVID-19 patients do not deteriorate after receiving plasma, and that adverse events associated with SARS-CoV-2 antibody-dependent mechanisms are low. More comprehensive studies are required to determine whether host responses to SARS-CoV-2 are protective or harmful. Although there is no registered antibody treatment available for COVID-19, there are two promising candidates. Regeneron’s polyclonal antibody cocktail (REGN-COV2) [73], which was used to treat President Trump [74], has been shown to reduce viral load and the need for further medical treatment in an ongoing phase II/III trial [75]. Bamlanivimab, a neutralizing monoclonal antibody cocktail produced by Eli Lilly, is also being tested on hospitalized COVID-19 patients and was recently granted Emergency Use Authorization by the FDA for COVID-19 treatment [76].

### 4.3. Global Distribution

If and when a vaccine against SARS-CoV-2 becomes available, there will be a need to manufacture and distribute sufficient quantities of the vaccine in order to immunize the global population. Many countries have pre-emptively secured millions of vaccine doses from pharmaceutical companies without the knowledge that these candidate vaccines will in fact work [77]. At this point it is unclear whether existing capacity is adequate for the provision of sufficient quantities of vaccines for the global population. The WHO has warned that protectionism might limit global availability, and that this will affect lower-to-middle income countries (LMICs) more severely [78]. Many routinely applied vaccines are being distributed to LMICs; however, there are many countries and areas where this is not the case, especially among poor and vulnerable communities. Maintaining low temperatures during shipment, especially to small towns and rural areas in LMICs, is an important challenge [79].

The African continent has limited capacity for vaccine manufacturing, even in countries such as South Africa. COVID-19 has demonstrated the need for such capacity, and it is hoped that this will be the catalyst for more countries, including LMICs, to develop vaccine manufacturing capability. To ensure access to sufficient quantities, South Africa is part of Covax, an initiative supported by the WHO that will pool resources for vaccine development and global distribution. South Africa has entered into three COVID-19 vaccine trials and is investing in its own capacity to produce and distribute a vaccine locally [80]. The South African government would need to subsidize a COVID-19 vaccine for it to be successful, since the majority of South Africans do not have medical insurance. It will be necessary to first vaccinate healthcare and frontline workers as well as individuals in high risk categories (including the aged and prison inmates), as has been suggested by the WHO [81].

### 4.4. Vaccine Hesitancy

A major obstacle to the success of a vaccine is vaccine hesitancy. Individuals opposed to vaccines do not consider them to be necessary and believe that the response of the immune system to the virus would be stronger if a person were to contract the illness naturally, and recover. Others feel that vaccines may not have been adequately tested and regulated before licensing. A significant increase in vaccine hesitancy was triggered following a 1998 Lancet publication, which suggested that the measles, mumps, and rubella (MMR) vaccine could lead to a higher incidence of developmental disorders in children, even though the research was discredited and the article retracted [82]. Those against COVID-19 vaccination are likely to oppose vaccinations in general and cite safety (including ADE of disease) as their main reason for hesitancy. However, a sizable number of people not usually opposed to vaccination are hesitant. This group appears to be concerned about the rapidity with which vaccine development is being approached and fear the long-term implications. It is important to reach these communities to obtain a better understanding of why they are uncertain or resistant to vaccination. Trust is critical if SARS-CoV-2 vaccines are to achieve their effect [3]. In contrast to the above, many of those who have personally experienced the impact of the pandemic and fear infection are anxiously waiting to be vaccinated when a vaccine becomes available [83].

## 5. Conclusions

Morbidity, mortality and the enormous socioeconomic consequences that have resulted from the COVID-19 pandemic necessitate the urgent development of a SARS-CoV-2 vaccine. Safety and efficacy at both pre-clinical and clinical stages are essential and are the reason for extended timelines normally required for vaccine development. The pandemic has seen the establishment of novel paradigms to fast-track candidate vaccine development in order to curb the spread of the virus. Although it is important to find a vaccine quickly, evidence-based decisions should remain the priority, and this takes time. Knowledge about SARS-CoV-2 and COVID-19 is accumulating rapidly; it is however unclear whether a vaccine will be able to completely eradicate this disease. Irrespective of the outcome, we will, at least in the short- to medium-term, need to find ways to manage life with the virus, and vaccination is a key requirement if we are to do so successfully.

## Figures and Tables

**Table 1:** Candidate vaccines in phase III of clinical evaluation.

Candidate	Platform	Vaccine Characteristics	Vaccine Targets	Developers	Clinical Stage
PicoVacc/CoronaVac	Inactivated	Inactivated SARS-CoV-2 alum adjuvant	Whole virus	Sinovac Research and Development Co., Ltd.	Phase I/II NCT04551547 Phase III NCT04456595
Inactivated SARS-CoV-2 vaccine	Inactivated	-	Whole virus	Beijing Institute of Biological Products/Sinopharm	Phase I/II ChiCTR2000032459 Phase III NCT04560881
Inactivated SARS-CoV-2 vaccine	Inactivated	-	Whole virus	Wuhan Institute of Biological Products/Sinopharm	Phase I/II ChiCTR2000031809 Phase III ChiCTR2000034780
BBV152	Inactivated	Inactivated whole virion	Whole virus	Bharat Biotech International Limited	Phase I/II CTRI/2020/07/026300 CTRI/2020/09/027674 NCT04471519 Phase III CTRI/2020/11/028976 NCT04641481
ChAdOx1 nCoV-19/AZD1222/Covishield	Non-replicating viral vector	Chimpanzee adenovirus containing the genetic sequence of the SARS-CoV-2 surface spike protein	Full-length S protein	University of Oxford/AstraZeneca	Phase I PACTR202005681895696 Phase I/II PACTR202006922165132 Phase II/III NCT04400838 Phase III ISRCTN89951424
Ad5-nCoV	Non-replicating viral vector	Ad5 vector	Full-length S protein	CanSino Biological Inc./Beijing Institute of Biotechnology	Phase I ChiCTR2000030906 Phase II ChiCTR2000031781 Phase III NCT04526990
Sputnik V	Non-replicating viral vector	Recombinant adenovirus type 26 (rAd26) and type 5 (rAd5) vectors carrying the gene for SARS-CoV-2 spike glycoprotein (rAd26-S and rAd5-S)	Full-length S protein	Gamaleya Research Institute/Health Ministry of the Russian Federation	Phase I/II NCT04436471 Phase III NCT04530396
Ad26.COV2.S	Non-replicating viral vector	Ad26 vector	Full-length S protein with 2 proline substitutions (K986P and V987P) and 2 mutations at furin cleavage site (R682S and R685G)	Janssen Pharmaceutical Companies	Phase I NCT04509947 Phase I/II NCT04436276 Phase II EUCTR2020-002584-63-DE Phase III NCT04505722
NVX-CoV2373	Protein subunit	Full length recombinant SARS-CoV-2 glycoprotein nanoparticle vaccine adjuvanted with Matrix M	Full-length S protein	Novavax	Phase I/II NCT04368988 Phase II NCT04533399 Phase III 2020-004123-16 NCT04611802
-	Protein subunit	Recombinant SARS-CoV-2 vaccine (CHO Cell)	RBD-dimer (residues 319–537 as tandem repeat)	Anhui Zhifei Longcom Biopharmaceutical/Institute of Microbiology, Chinese Academy of Sciences	Phase I NCT04445194 Phase I/II NCT04550351 Phase II NCT04466085 Phase III ChiCTR2000040153
mRNA-1273	RNA	LNP-encapsulated mRNA encoding the surface spike protein	Full-length S protein with 2 proline substitutions (K986P and V987P)	Moderna/NIAID	Phase I NCT04283461 Phase II NCT04405076 Phase II/III NCT04649151 Phase III NCT04470427
BNT162b2	RNA	LNP nucleoside-modified mRNA encoding an optimized SARS-CoV-2 RBD antigen	Full-length S protein with 2 proline substitutions (K986P and V987P)	BioNTech/Fosun Pharma/Jiangsu Provincial Center for Disease Prevention and Control/Pfizer	Phase I/II 2020-001038-36 ChiCTR2000034825 NCT04537949 Phase III NCT04368728

References [9,28]. CHO: Chinese hamster ovary; LNP: lipid nanoparticle; mRNA: messenger RNA; NIAID: National Institute of Allergy and Infectious Diseases; RBD: receptor binding domain; RNA: ribonucleic acid; S: spike.
